# Fatigue Cracking Characteristics of Ultra-Large Particle Size Asphalt Mixture Under Temperature and Loading Using Digital Image Correlation Techniques

**DOI:** 10.3390/ma18071475

**Published:** 2025-03-26

**Authors:** Tian Tian, Yingjun Jiang, Yong Yi, Chenliang Nie, Changqing Deng

**Affiliations:** 1 School of Civil Engineering and Architecture, Xinjiang University, Urumqi 830039, China; 2 Xin Jiang Key Laboratory of Building Structure and Earthquake Resistance, Xinjiang University, Urumqi 830047, China; 3 Key Laboratory for Special Area Highway Engineering of Ministry of Education, Chang’an University, South 2nd Ring Rd., Middle Section, Xi’an 710064, China; jyj@chd.edu.cn (Y.J.); yyong@chd.edu.cn (Y.Y.); 2020221248@chd.edu.cn (C.N.); changqingdeng@chd.edu.cn (C.D.); 4 School of Civil and Architectural Engineering, Yangtze Normal University, Fuling, Chongqing 408100, China; 5 College of Urban and Rural Construction, Shaoyang University, Shaoyang 422000, China

**Keywords:** ultra-large particle size asphalt mixture (LSAM-50), fatigue life prediction, temperature–stress coupling, digital image correlation (DIC), crack propagation, splitting fatigue test (SFT)

## Abstract

This study quantitatively investigates the fatigue cracking behavior of ultra-large particle size asphalt mixture (LSAM-50) under coupled temperature and stress effects. Fatigue tests were conducted across temperatures ranging from −15 °C to 35 °C and stress levels (0.3–0.9 of splitting tensile strength), with crack evolution tracked in real time using digital image correlation (DIC). Key parameters, including main crack length, crack density, curvature, fractal dimension, and strain, were analyzed to characterize crack propagation. Results revealed a three-stage process: initiation, development, and acceleration to failure. Increasing temperature or stress level accelerated horizontal/vertical displacement rates, main crack expansion, and strain accumulation, while reducing crack density and fractal dimension. A fatigue prediction model, Lg*N* = 9.741 − 1.213Lg*ε* − 0.017*T* − 1.579*S* (*R*^2^ = 0.954), was established, linking fatigue life (*N*) to strain (*ε*), temperature (*T*), and stress level (*S*). This model enables precise fatigue life estimation under varying environmental conditions. For instance, the model predicts a 60% reduction in fatigue life when temperature rises from 15 °C to 35 °C at *S* = 0.7, highlighting its utility in material selection for climate-resilient infrastructure, offering a critical tool for optimizing LSAM-50 in pavement design. By integrating DIC-derived crack metrics and mechanistic insights, this work not only enhances understanding of the fatigue cracking behavior of LSAM-50 but also provides valuable insights for the design and optimization of materials under varying environmental conditions.

## 1. Introduction

Flexible subbases have garnered significant scholarly interest, owing to their benefits such as low-temperature cracking resistance, strong interlayer adhesion, minimal modulus differential between layers, and the absence of reflective cracking [1]. Nonetheless, the prevalent flexible subbase materials are often associated with high engineering costs, a factor that hinders the widespread adoption of full-depth asphalt pavement structures in China [2]. In response to this challenge, our research team has developed an asphalt mixture with ultra-large aggregate particles, having a nominal maximum size of 53 mm (LSAM-50) [3,4]. This novel mixture exhibits enhanced strength, rut resistance, and cost-effectiveness, positioning it as a potential new flexible base material. Studies [5,6] indicate that fatigue cracking is a predominant failure mode in flexible base asphalt pavements, a condition influenced by factors such as the pavement’s structural configuration and the thickness of the structural layers. The structural design of flexible base asphalt pavements hinges on this fundamental principle. Consequently, the investigation into the fatigue performance of LSAM-50 material has emerged as a pivotal research area.

One concern is that conventional asphalt mixtures are typically fatigue tested at temperatures in the range of 10 °C to 20 °C [7]. In contrast, LSAM-50’s flexible base layer is used at temperatures beyond this range [8]. Previous research [4] has identified LSAM-50 as a quintessential viscoelastic material [9,10], with its mechanical attributes heavily influenced by ambient temperatures and loading conditions. As such, the impact of both temperature and load must be meticulously considered in the fatigue performance analysis of LSAM-50 materials. Scholars have delved into the temperature’s influence on asphalt mixture fatigue. For instance, Son et al. [11] explored the fatigue fracture characteristics of SMA-13 mixtures at −12 °C, 0 °C, and 10 °C through semi-circular bending tests. Al-Khateeb et al. [12] examined the fatigue performance of asphalt mixtures at different loading frequencies at 20 °C and 30 °C. Ehsan et al. [13] assessed the fracture energy of fiber-modified asphalt mixtures at −18 °C and 25 °C. Cheng et al. [14] compared the fatigue life of AC-16 mixtures at 15 °C to 40 °C using both four-point bending and indirect tensile fatigue tests. Liu et al. [15] simulated the temperature stress change rule as the internal temperature of porous asphalt mixture decreased from 25 °C to −25 °C. Lastly, Luo et al. [16] analyzed the thermal stress changes of asphalt mixtures within the temperature ranges of −20 to −10 °C and −10 to 0 °C through thermodynamic constraint tests. These comprehensive studies have conclusively demonstrated the profound impact of temperature on the fatigue performance of asphalt mixtures. They have also provided invaluable insights into the fatigue study of LSAM-50, prompting us to delve into the fatigue performance of this material across varying temperatures during the preliminary phase. Consequently, a fatigue life prediction model for LSAM-50 was developed using machine learning techniques [3]. The fatigue life, a straightforward and intuitive metric, has emerged as a standard for assessing the fatigue characteristics of asphalt mixtures [17]. However, fatigue cracking, a formidable challenge in the analysis of mixture properties, exhibits a complex fatigue damage mechanism. To enhance the evaluation of fatigue life and further analyze fatigue cracking characteristics, additional evaluation parameters are indispensable. Notably, the quantification and evaluation of cracks in LSAM-50 remain an unresolved issue.

The advent of cutting-edge scientific research equipment has revolutionized the study of asphalt mixture fatigue damage evolution. Advanced technologies such as Scanning Electron Microscopy (SEM), Computed Tomography (CT), and digital image correlation (DIC) have become staples in this field [18,19,20]. Notably, researchers have posited that the self-healing capabilities of asphalt binders over time significantly influence the actual cracking patterns of asphalt mixtures [21]. Furthermore, the frequency of testing is shown to impact the accurate characterization of crack resistance. Traditional SEM and CT equipment, while capable of imaging, necessitate the interruption of testing to remove samples for scanning, thereby precluding real-time crack monitoring. In contrast, DIC offers a dynamic approach to capturing displacement and strain fields during testing, making it a preferred method for material cracking evaluation. Hill et al. [22] leveraged DIC to explore the effects of loading rate, temperature, and recycled materials on the fracture response of asphalt mixtures. Similarly, Jiang et al. [23] investigated the correlation between fatigue cracking damage and average tensile strain, utilizing DIC to assess the strength and fatigue properties of asphalt mixtures through displacement development and energy consumption during cyclic loading. Yuan et al. [24] employed DIC to extract displacement and strain fields under semicircular bending tests. Consequently, DIC providing valuable assistants into the fatigue cracking displacement evolution and crack extension behavior of asphalt mixtures under dynamic loading. This technique allows for the determination of multiple cracking parameters, aiding in the comprehensive characterization of material crack resistance.

This study aims to quantitatively evaluate the fatigue cracking behavior of LSAM-50 under combined temperature and stress effects using DIC and establish a predictive model for material fatigue life. In view of this, LSAM-50 was taken as the research object, and the fatigue life at different temperatures and stress levels was tested by splitting fatigue test (SFT) using VVTM molded specimens. The evolution patterns of both horizontal and vertical displacements during the fatigue cracking process of LSAM-50 were meticulously analyzed through real-time monitoring with DIC. Parameters such as the main crack length, crack density, crack curvature, fractal dimension, and strain were meticulously calculated at the range of damage, then the strain cloud diagrams for the crack extension phase were generated. The impact of temperature and stress levels on the cracking resistance of LSAM-50 were quantitatively evaluated. Ultimately, a comprehensive fatigue prediction model for LSAM-50 under the combined influence of temperature and stress levels was proposed. This research significantly contributes to the comprehensive characterization of the fatigue cracking properties of LSAM-50 and offers valuable insights for material design.

## 2. Materials and Methods

### 2.1. Materials

#### 2.1.1. Raw Materials

Table 1 lists the technical specifications of Donghai 70#A Class highway petroleum asphalt (produced by Shandong High Speed Material Group Company, Ji’nan, China) used in this study. In addition, the aggregates and mineral powders used in this study were crushed from limestone, and originated from Ji’nan, Shandong, China and Xi’an, Shaanxi, China respectively. And, the raw materials technical indexes all meet the relevant requirements, which are omitted here.

#### 2.1.2. Gradation

**Figure 1:**
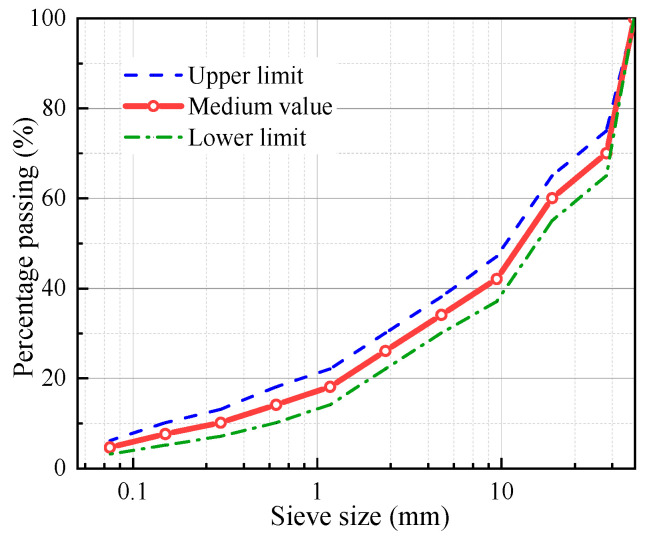
LSAM-50 gradation curve.

Figure 1 illustrates the LSAM-50 gradation range, with a maximum nominal particle size of 53 mm, as determined by preliminary research [3,4]. In this study, the median of the LSAM-50 gradation range was employed. This selection ensures that the study’s findings are representative of the broader asphalt mixture population.

#### 2.1.3. Asphalt Mixture

The technical properties of LSAM-50 mixture used for this study [3,4] are shown in Table 2.

### 2.2. Specimen Preparation

The Marshall method stands as the predominant technique for indoor specimen formation in asphalt mixtures, a practice extensively employed in current research. Nevertheless, the escalating weight of construction equipment has led to a discrepancy in mechanical strength between Marshall-prepared specimens and field core samples, with a correlation rate of less than 70% [4]. To address this, our research group has developed the Vertical Vibratory Compactor (VVC), (Tianjin Dongzheng measurement and control Technology Development Co., Ltd., Tianjin, China), which, grounded in the principles of vibratory rollers, effectively replicates the compaction dynamics at construction sites [25]. This innovative equipment achieves a remarkable correlation of 92% between its formed specimens and the mechanical strength of core samples, while minimizing aggregate crushing. Consequently, the vertical vibration test method (VVTM) has been adopted for specimen formation in this study.

**Figure 2:**
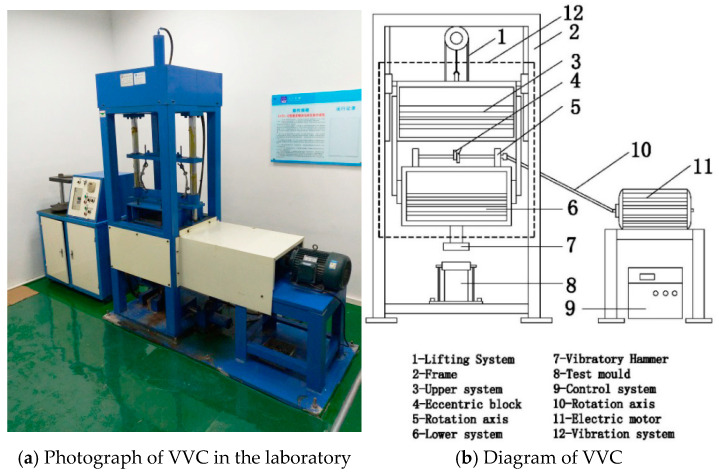
Schematic of VVC.

Figure 2 shows the specific structure of the instrument. In this study, the LSAM-50 cylinder specimen with a size of *φ*200 mm × *h*160 mm was formed by a VVC. The VVC parameters meticulously chosen to align indoor-formed specimens with the materials of actual construction sites are as follows [4]: an operating frequency of 40 Hz, a nominal amplitude of 1.2 mm, an upper system weight of 1.2 kN, and a lower system weight of 90 kN.

### 2.3. Test Methods

#### 2.3.1. Splitting Fatigue Test

**Figure 3:**
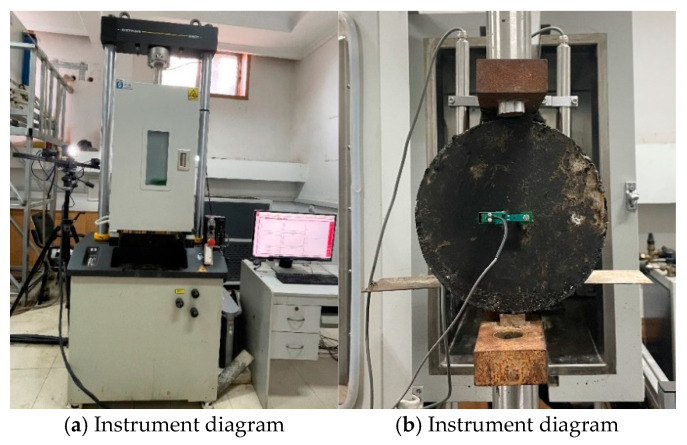
SFT diagram.

It should be noted that before SFT, the split tensile strength was tested as a fatigue test parameter in accordance with the Chinese standard JTG E20-2011 T0716 [26], and the calculation formula is shown in Equation (1). The equipment used by the experimenters is shown in Figure 3.

As depicted in Figure 3, SFT employed an electrohydraulic servo fatigue testing machine (MTS 8800), (Instrong (Shanghai) Test Equipment Trading Co., Ltd., Shanghai, China), which was equipped with an environmental chamber produced by the same company. To accurately measure and monitor the temperature of the specimens, the temperature chamber was fitted with a reference specimen, which featured two Linear Variable Differential Transformers (LVDTs), (Instrong (Shanghai) Test Equipment Trading Co., Ltd., Shanghai, China). To minimize temperature errors during the test, LSAM-50 cylinder specimens, insulated to a specific temperature, were placed inside the environmental chamber of the MTS, for an additional insulation period of 0.5 to 1 h prior to the test. This is due to the asphalt mixture’s sensitivity to temperature fluctuations. Real-time monitoring of the temperature change within the chamber commenced once the calculated temperature change coefficient was less than 5%.

The key test parameters utilized in the SFT are presented in Table 3. Notably, a 120-s preloading phase (20% of the failure load) was applied prior to fatigue testing. This preload served two purposes: ensuring uniform contact between specimens, fixtures, and supports, and verifying the proper operational status of the testing apparatus.

**Figure 4:**
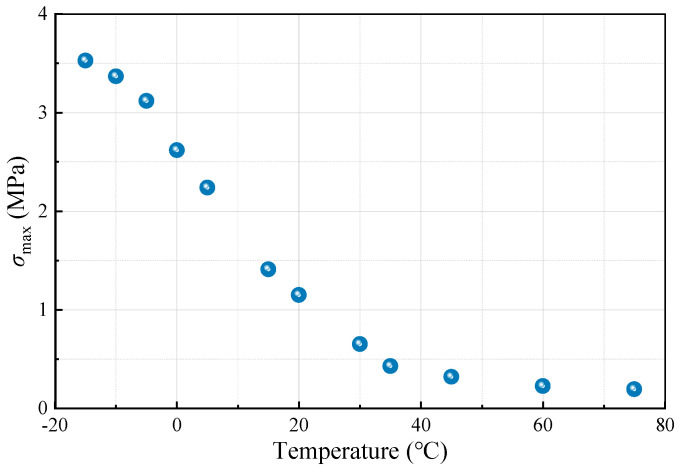
Values of *σ*_max_ for LSAM-50.

Significantly, the stress level *S* can be defined as: *S* = *σ*_max_/*σ*_S_, where *σ*_max_ denotes the maximum cyclic stress, and *σ*_S_ represents the ultimate splitting strength of LSAM-50. The values of *σ*_max_ for LSAM-50 under different temperatures are presented in Figure 4. For each test group, four parallel specimens were prepared, and the test results were reported as representative values at a 95% confidence level with a coefficient of variation less than 10%.

#### 2.3.2. Digital Image Correlation

**Figure 5:**
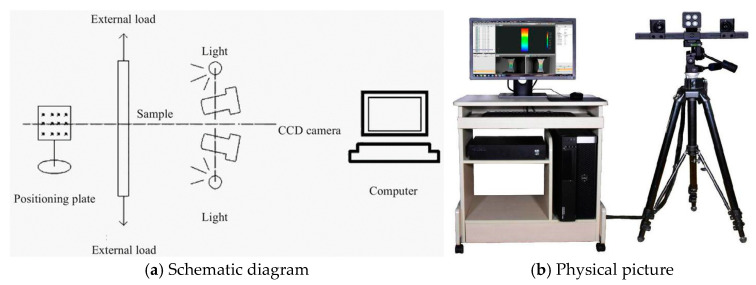
XTDIC system.

The three-dimensional full-field strain measurement system (XTDIC) serves as a pivotal tool for real-time monitoring of surface cracks, displacements, and strains in fatigue tests on specimens. This optical, non-contact deformation measurement system encompassing an adjustable measuring head, a control box, and a high-performance computer, as depicted in Figure 5.

**Figure 6:**
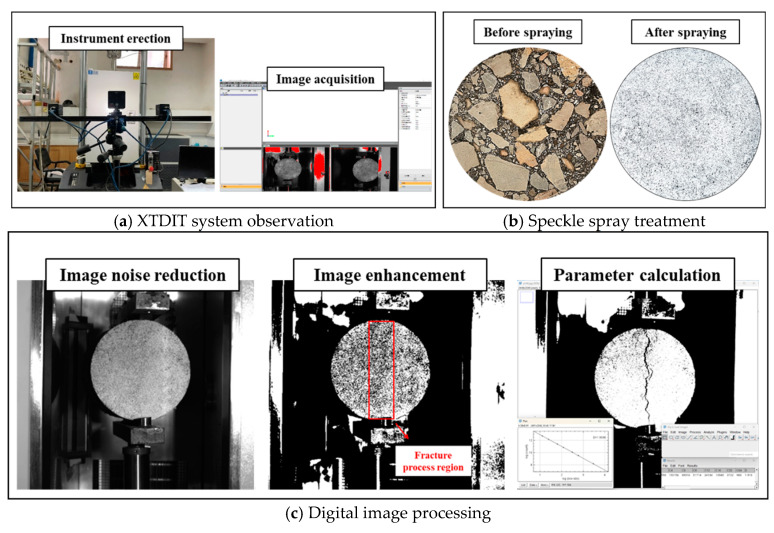
Digital image correlation technology.

To enhance the precision of digital image analysis, the cross-sectional area of the LSAM-50 specimen is meticulously pre-processed with scatter spraying prior to testing. Subsequently, the captured images undergo noise reduction and enhancement through MATLAB R2022a software, ensuring optimal image quality. The entire digital image processing workflow is illustrated in Figure 6.

This study quantified fatigue cracking severity in LSAM-50 using parameters including principal crack length, crack density, crack curvature, and fractal dimension. The main crack length refers to the total length of the crack within the vertical projection plane. The crack density is calculated as the ratio of the crack length to the area of the fracture process zone, which, for the LSAM-50 cylindrical specimen, spans approximately 97 cm^2^, as shown in Figure 6c. The crack curvature is the ratio of the total length of the main crack to its chord length within the fracture process zone. The fractal dimension [27], a powerful tool in fractal analysis, is utilized to define the shape of the crack boundary or contour, reflecting the degree of fragmentation or irregularity at various scales. These parameters were meticulously obtained from digital images through real-time monitoring of the specimen’s fatigue loading process using the XTDIC system. Subsequently, the evaluation parameters were calculated using the Frac Lac plug-in within the Image J2 software, as depicted in Figure 6c. The fatigue damage process of LSAM-50 scatter specimen during fatigue loading was monitored in real time by XTDIC system. The acquired images are processed with image input, digitization, image noise reduction, image enhancement, feature information extraction, and output results. Combined with the full-field strain measurement method [24] to correct the strain accuracy, the LSAM-50 splitting fatigue strain cloud was obtained.

## 3. Results and Discussion

### 3.1. Equivalent Fatigue Life Calculation

**Figure 7:**
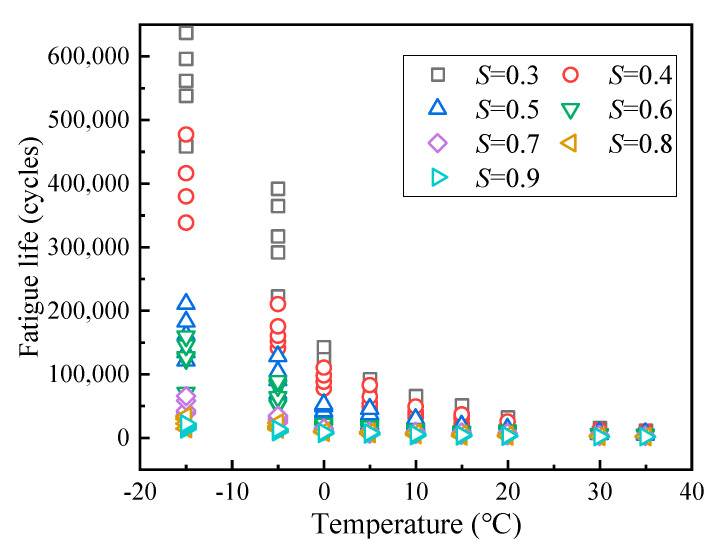
SFT results of LSAM-50.

The fatigue life of LSAM-50 is shown in Figure 7.

**Figure 8:**
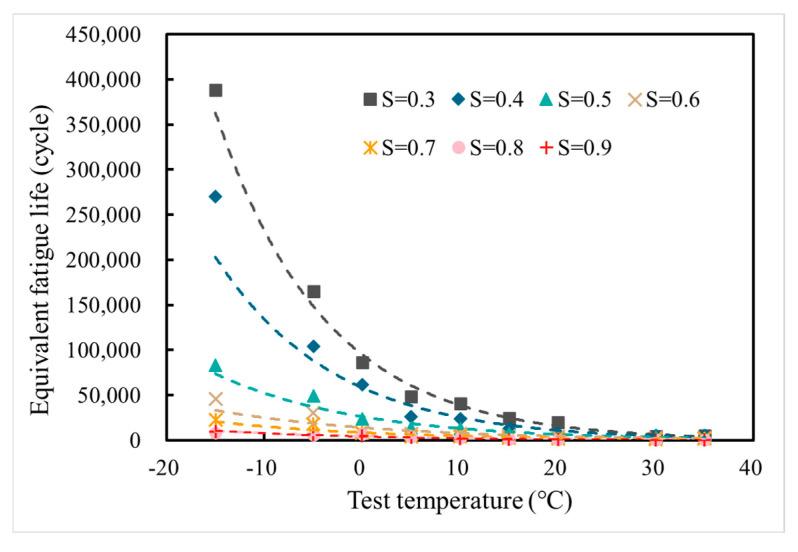
Equivalent fatigue life of LSAM-50 at 5% failure probability.

As shown in Figure 7, LSAM-50 fatigue life is inversely proportional to both temperature and stress level. Notably, the coefficients of variation for the fatigue life test results range from 6% to 26%, with an average of 19% following the elimination of outliers. However, the overall experimental results exhibit a degree of scatter that hinders subsequent analytical processes. The Weibull distribution, renowned for its ease in inferring distribution parameters from probability values, is widely utilized in reliability engineering data processing. This method is grounded in the theoretical underpinnings of reliability analysis, adheres to the physical principles of material fatigue, and accurately reflects the distribution law of design parameters [28]. Thus, the Weibull distribution has been employed to calculate the equivalent fatigue life [3,28] of LSAM-50 at a 5% failure probability, with the results presented in Figure 8.

From Figure 8, the equivalent fatigue life decreases gradually with increasing temperature and the rate of change slows down, which approximates the exponential trend. N.A. Hassan proposed that stiffness has an important effect on the fatigue life of asphalt mixtures. When the temperature decreases, the mixture stiffens, stiffness increases, strength increases, and fatigue life increases. Asphalt mixture is a typical temperature sensitive material. With increasing temperature, asphalt mixtures gradually change from elastic characteristics exhibited at low temperatures to viscoelasticity–plasticity. And the mobility increases, and the bonding effect between aggregate and asphalt binder is gradually weakened. Ultimately, the fatigue life of the mixture is reduced, which is similar to the results of the previous bending fatigue [3].

### 3.2. Fatigue Cracking Displacement Evolution

#### 3.2.1. Vertical Displacement

**Figure 9:**
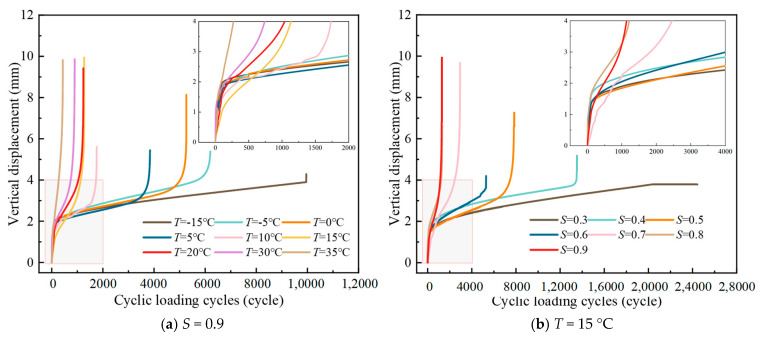
Curve of vertical displacement~cyclic loading cycles.

The curves of vertical displacement of LSAM-50 with the number of cyclic loadings are shown in Figure 9.

**Figure 10:**
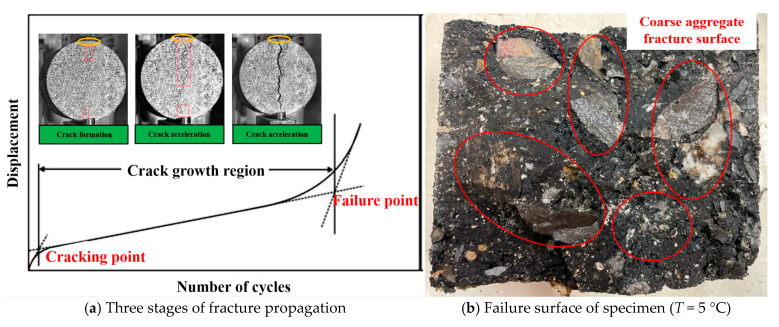
Fatigue cracking diagram of LSAM-50.

From Figure 9, it is evident that the vertical displacement exhibits a distinct three-phase progression as the cyclic actions accumulate. Analyzing the tangent lines of the curves within each phase reveals two pivotal intersection points, which delineate the three stages of the crack propagation process: initiation, growth, and the acceleration towards destabilization, as depicted in Figure 10a. During the initial loading phase, displacement escalates rapidly, followed by a nearly linear increase, culminating in a phase where the specimen is on the brink of destruction, marked by a significant acceleration in displacement.

From Figure 9, it is known that with the increase of temperature or stress level, the rate of vertical displacement development increases and the fatigue life appears to be reduced to different degrees. The analysis of the damage surface of the fatigue specimen at low temperatures depicted in Figure 10b reveals that the LSAM-50 specimen’s strength is predominantly derived from its coarse aggregates. Post-temperature decrease, the asphalt binder’s hardness increased, and its adhesion with the aggregates intensified [28,29]. Consequently, the specimen damage predominantly presented itself as coarse aggregate fractures. An enhancement in the mixture’s fatigue life could potentially be achieved by improving the quality of the aggregates.

Ghazi et al. [12] concluded that the relationship between temperature and fatigue life depends on the loading mode. De Freitas et al. [30] concluded that fatigue life is highly dependent on temperature in stress-controlled loading test mode. Holding other factors constant, decreasing the temperature increases the fatigue life and increasing the temperature leads to top-down fatigue cracking and decreases the fatigue life, which is similar to the experimental results obtained in this study in the stress-controlled mode. On the other hand, it has been suggested in the literature [31,32] that fatigue life increases with increasing temperature (from 20 °C to 30 °C) when strain-controlled loading mode is used. Therefore, in addition to the loading mode, it is considered that the difference in the test pattern may involve the mix type, gradation, and so on.

The local enlarged diagram shows that the initial vertical displacement mostly increases sharply by 1~2 mm at the beginning of the loading stage. This observation suggests that the test preload may be inadequate, leading to insufficient contact between the specimen and the fixture. Alternatively, it could be that the fine aggregates on the specimen’s surface are being subjected to compressive forces from the indenter at both ends, resulting in extruded deformation, as depicted in Figure 10a. With the growth of the number of cycles, the phenomenon of delayed or stagnant displacement expansion occurs in some sections, indicating that the internal cracks of the specimen are hindered by the coarse aggregate, which produces a “hysteresis effect”. In addition, part of the data is affected by the loading of sinusoidal wave, and there will be a “wave section”.

#### 3.2.2. Horizontal Displacement

**Figure 11:**
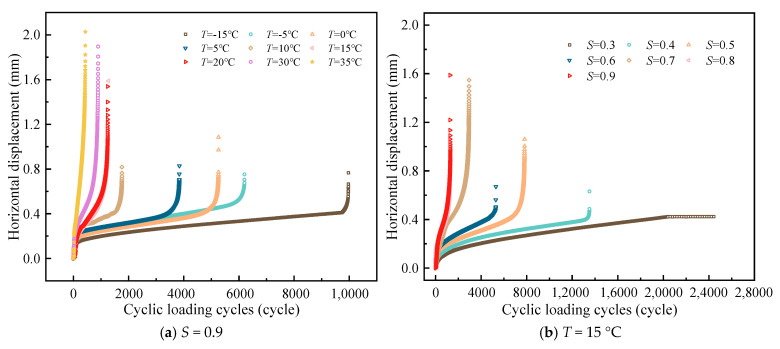
Curve of horizontal displacement ~ cyclic loading cycles.

The variation curves of horizontal displacement of LSAM-50 with the number of cyclic loadings are shown in Figure 11.

As illustrated in Figure 11, the general trend of horizontal displacement aligns with that of the vertical displacement curve as the number of cyclic actions increases. However, the variability of horizontal displacement under various influencing factors is notably more pronounced, and there is an overall reduction in horizontal displacement. This phenomenon could be attributed to the high precision of the extensometer, which is positioned only in the area of the specimen surface fracture process (refer to Figure 3). This positioning mitigates the interference from experimental errors, such as uneven contact between the specimen and the fixture due to insufficient preload, as well as local deformation of the specimen surface at the indenter contact point under excessive preload or stress levels. Furthermore, the horizontal displacement curve is less susceptible to exhibiting a “wave-like” pattern, suggesting that the test surface is largely unaffected by sinusoidal loading interference.

In summary, the collection of vertical displacement during SFT is a straightforward process. Nevertheless, the use of sinusoidal loading methods and the significant deformation at the contact point between the specimen and the indenter may compromise the accuracy of displacement data. Some researchers [33] have posited that horizontal displacement offers a more comprehensive reflection of the fatigue cracking displacement evolution. However, it has been observed that certain specimens are impacted by internal voids and other factors, leading to cracks that do not traverse the indenter contact points at the specimen’s upper and lower ends. In practical applications, the extensometer is typically set up in the fracture process area, with a limited range, which consequently results in the loss of horizontal displacement data. Therefore, a comprehensive understanding of the fatigue cracking displacement evolution in asphalt mixtures necessitates the integration of both vertical and horizontal displacements.

### 3.3. Crack Expansion Behavior Analysis

#### 3.3.1. Main Crack Length

**Figure 12:**
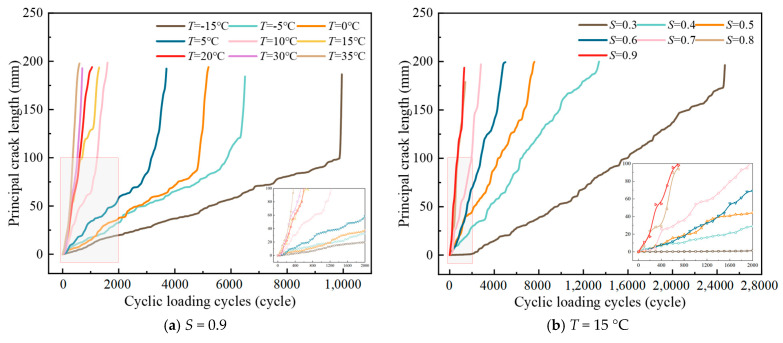
Curve of principal crack length~cyclic loading cycles.

The variation curves of the main crack length of LSMA-50 with the number of cyclic loadings are shown in Figure 12.

From Figure 12, LSAM-50 fatigue specimens show cracks after accumulating fracture energy in about 100~2000 cycles. Subsequently, the crack expansion rate increases sharply and tends to stabilize. Finally, the crack expansion rate increases rapidly until final damage occurs. A notable aspect of the crack expansion process is the occurrence of the “crack bending” phenomenon, where the expansion rate is notably lower than during the initial stages. This is predominantly attributed to the fact that cracks propagate predominantly along the path of least energy within the material, specifically the shorter route between the asphalt mortar and the aggregate surface. The slower the rate of crack propagation in the second stage of the LSAM-50 specimens, the greater their fatigue life. This could be attributed to the fact that lower temperatures enhance the strength of the asphalt binder to a certain extent, thereby reinforcing the “crack bending” effect and, consequently, the fatigue life of the specimens. At lower stress levels, particularly for asphalt mixtures under normal low-temperature conditions, energy is primarily dissipated during the fracture process. In contrast to non-plastic deformation, while the crack wall may open more during low-stress cycling, at higher stress levels, the time required for material damage is significantly reduced, the critical crack size for fatigue damage is reached sooner, and the specimens exhibit a reduced fatigue life. Drawing upon the toughening theory of ceramic matrix composites [34], the presence of large particle size aggregates functions as a pin, effectively locking the crack front and preventing further crack propagation.

#### 3.3.2. Crack Density

**Figure 13:**
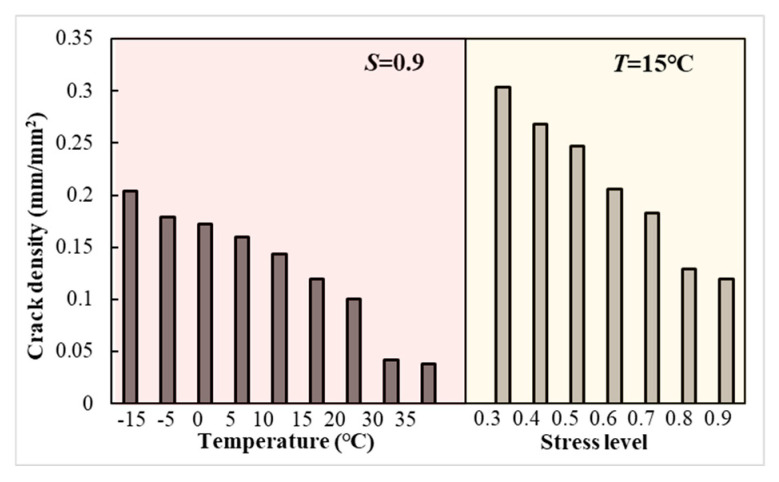
Crack density of specimen at failure.

The crack densities of LSAM-50 specimens at damage for different temperatures and stress levels are shown in Figure 13.

**Figure 14:**
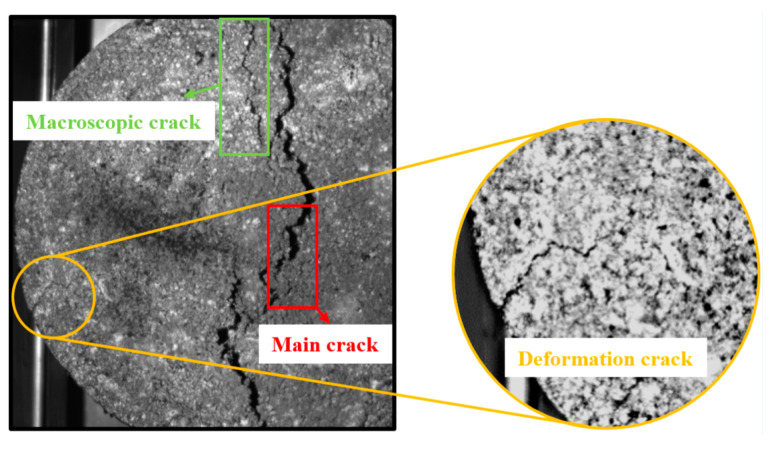
Different crack types on the surface of the specimen.

In Figure 13, it becomes evident that as temperature (or stress level) escalates, the crack density diminishes during specimen damage. There is a similarity between this pattern and the findings of Radeef et al. [35] regarding the increase in fatigue life and decrease in crack density of rubberized asphalt mixtures. The crack density is defined as the ratio of crack length to the area of the fracture process zone, and an increase in this ratio signifies the development of a network of cracks under applied loads. The rate at which cracks propagate is influenced by the type of crack, and the various crack types present on the fatigue specimen surface are depicted in Figure 14.

Figure 14 illustrates that deformation cracks are minute fissures that appear on the specimen’s surface. These cracks are unable to penetrate the matrix due to microstructural barriers and are not associated with the fatigue life of the specimen. Conversely, the macroscopic crack density is linked to the energy consumed during the plastic deformation of the specimen, which has a more profound impact on the fatigue life of asphalt mixtures. Al-Qadi et al. [36] concluded that the fracture energy is insufficient to differentiate the fatigue life of asphalt mixtures. The fracture energy is a function of the ductility and strength of the material, and if the peak load of the material is high, it may make up for the lack of ductility, so the fatigue life of the mixture cannot be estimated based on the fracture energy method alone. The fracture mechanics theory [19,20] proposes that the cumulative stress near the crack tip must be greater than the allowable stress as the crack expands, and fracture occurs when the applied stress is sufficient to break the atomic bonds of the solid. For LSAM-50, the cumulative stress required for crack expansion refers to the stress required to overcome the bond between the aggregate and the asphalt binder, which explains the inverse relationship presented by the aforementioned crack density and fatigue life. In addition, LSAM-50 tested at low stress levels had higher crack densities than at high stress levels, this result may be due to the increased specimen creep deformation due to the loading time [36].

#### 3.3.3. Crack Curvature

**Figure 15:**
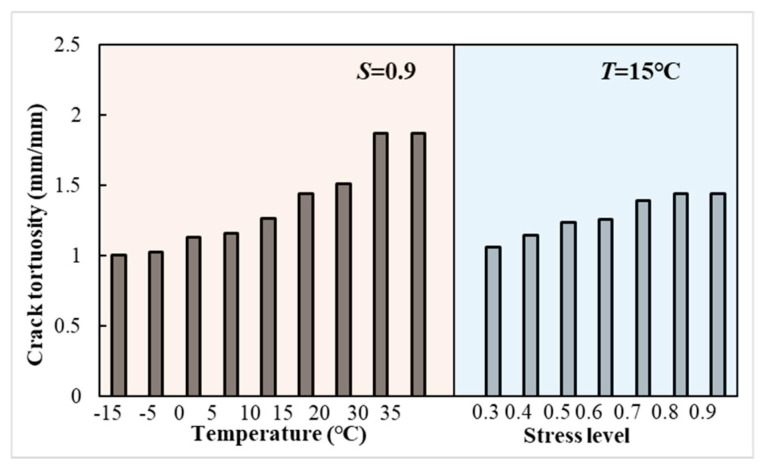
Crack tortuosity of specimen at failure.

The crack curvature of LSAM-50 specimens at damage for different temperatures and stress levels is shown in Figure 15.

Figure 15 illustrates a significant trend: the curvature of cracks escalates as the temperature (or stress level) rises during the phase of specimen damage. This observation aligns with established principles, as the curvature of a crack is the weakest link in its propagation, and the fatigue life of the specimen is positively correlated with this curvature. As temperature increases, the bond between asphalt binder and aggregate weakens, providing more avenues for cracks to extend and deflect. Conversely, a decrease in temperature enhances the stiffness of the asphalt mixture, diminishing its non-uniform properties [37,38]. The LSAM-50’s strength is predominantly derived from its large aggregate, which contributes to reduced crack flexure at the point of specimen damage. As stress levels increase, so does the fatigue load, leading to a greater number of relative weak paths within the specimen. This exacerbates crack curvature and diminishes the fatigue life. Furthermore, the inclusion of large-grained aggregate in LSAM-50 expands the fracture process zone compared to conventional asphalt mixtures, leading to heightened energy consumption during the crack expansion phase and an extended fatigue life [3,4].

#### 3.3.4. Fractal Dimension

**Figure 16:**
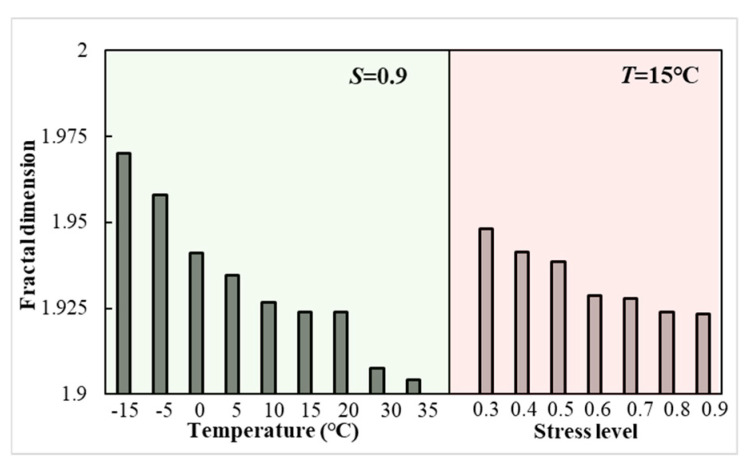
Fractal dimension of specimen at failure.

The fractal dimension of LSAM-50 specimens at damage for different temperatures and stress levels is shown in Figure 16.

**Figure 17:**
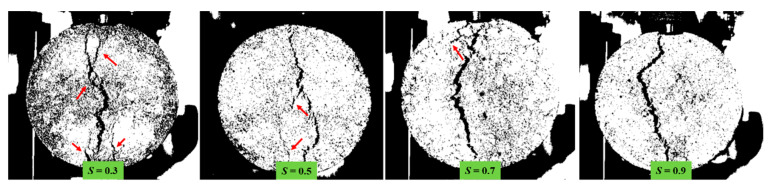
Surface crack shapes of LSAM-50 specimen under different stress levels (*T* = 15 °C).

From Figure 16, the fractal dimension of the specimen at damage decreases with increasing temperature (or stress level). The higher the fractal dimension, the more cycles need to be loaded before damage. Temperature affects the bonding properties of asphalt mixtures, warming leads to a decrease in the resistance of the material to crack expansion, the fractal dimension of the specimen damage at low temperatures is the largest, the highest fatigue life. This phenomenon can be attributed to the non-uniform properties of asphalt mixtures, as suggested by reference [39,40]. Specifically, at lower temperatures, asphalt mixtures experience a decrease in fluidity, an increase in stiffness, a reduction in non-uniformity, and an enhancement in bonding properties. These changes alter the pattern of crack expansion within the mixture, thereby increasing the proportion of aggregate strength within the overall mix strength. Furthermore, the presence of larger aggregates within the mixture amplifies the deformation potential within the fracture process zone, thereby delaying the LSAM-50 crack development stage and extending the fatigue life. Additionally, the fractal dimension at the time of specimen damage under high stress levels is generally lower than that observed at low stress levels. This discrepancy is primarily due to the viscoelastic–plastic properties of asphalt mixtures, which are time-dependent. A reduction in stress levels leads to asphalt mixture energy dissipation manifesting as creep deformation rather than fracture deformation, as evidenced by the greater deformation of the crack path at lower stress levels of LSAM-50, as depicted in Figure 17.

#### 3.3.5. Strain

**Figure 18:**
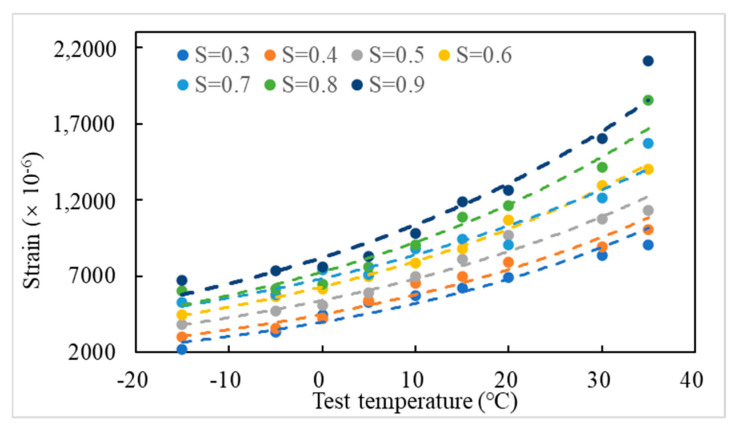
Fracture fatigue failure strain of LSAM-50.

**Figure 19:**
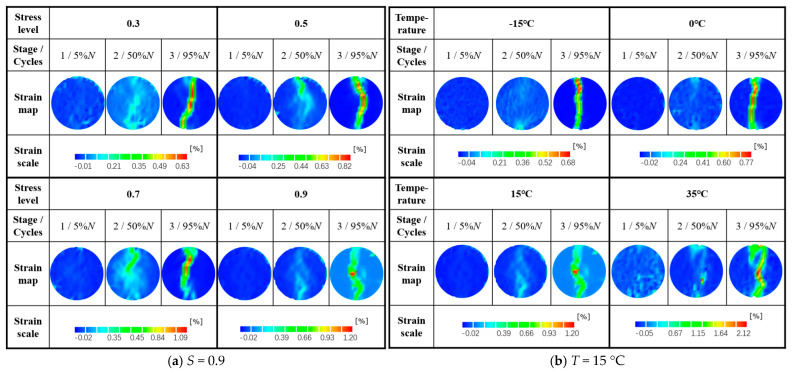
Strain map at different stages of surface crack propagation.

The LSMA-50 horizontal damage strains at different temperatures and stress levels are shown in Figure 18. Partially, the three-stage strain cloud for crack extension is shown in Figure 19.

Figure 18 shows that with the increase of temperature (or stress level), the strain of specimen damage increases. Figure 19 shows that there is a strain concentration phenomenon at the contact point between the end of the specimen and the fixture, which coincides with the conclusion that “the fine aggregate on the surface of the specimen is easily squeezed by the force of the indenter at both ends”. Comparing the strain cloud diagram of 50% loading times, with the increase of temperature (or stress level), the crack penetration rate increases and the deformation crack decreases at this stage, which is consistent with the aforementioned law related to the crack expansion rate. Reducing the temperature enhances the critical stress strength of the weakest bonding path of the asphalt mixture, which improves the cracking resistance of the mixture. Comparing the strain cloud plots at 95% loading times, the higher the temperature, the greater the LSAM-50 fracture process zone and crack curvature, which reflects the decrease in the bond between asphalt binder and aggregate with increasing temperature. More macroscopic cracks were extended around aggregate particles under loading, which coincides with the aforementioned law related to crack curvature.

### 3.4. Fatigue Life Prediction Modeling

The fatigue equation of materials serves as the foundation for the antifatigue design of pavements. Thus, it is of great significance whether the form of the equation can reveal the internal mechanism of fatigue failure in asphalt pavements under the repeated action of traffic loads and environmental factors. Most existing material fatigue equations adopt the form of *N*~*S* or *N*~*σ* [4], and scarcely consider the effect of environmental factors, especially temperature. Therefore, in this section, a fatigue equation for LSAM—50 under the coupled action of temperature and load will be established, laying a foundation for the fatigue life prediction and antifatigue design of LSAM—50 pavements.

In the study, Lg*ε*, *T* and *S* were employed as independent variables, while Lg*N* served as the dependent variable. A series of multiple linear regressions were conducted using IBM SPSS Statistics 26, with the outcomes presented in Table 4, Table 5 and Table 6. Here, *T* and *S* denote temperature and stress levels, respectively, and ε represents the destructive strain corresponding to the equivalent fatigue life *N* at 5% failure probability.

Lg*N* = 9.741 − 1.213Lg*ε* − 0.017*T* − 1.579*S* (1)

where *N* is the fatigue life of LSAM-50 (cycle); *T* stands for temperature (° C); *S* is stress level; and *ε* represents the failure strain corresponding to the *N* with 5% failure probability, ×10^−6^.

**Figure 20:**
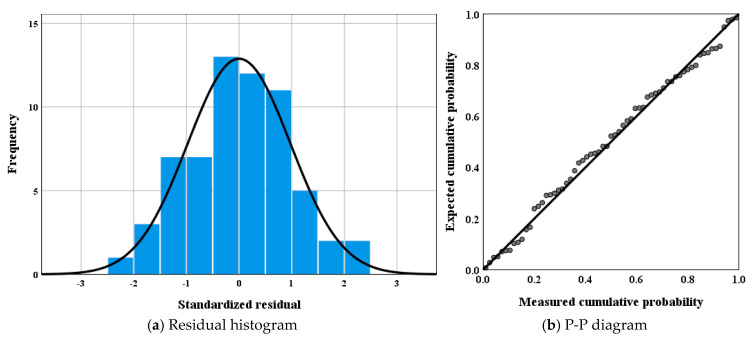
Model error verification diagram.

The residual histogram and P-P diagram of the model are shown in Figure 20.

In Figure 20, the histogram of residuals adheres to a normal distribution, with a mean value approaching zero. The standard deviation is nearly equivalent to that of a standard normal distribution. Moreover, the points on the P-P plot align approximately in a straight line, suggesting that the linear regression has been achieved under the condition of normality. This indicates that Equation (1) can effectively describe the functional relationship between the independent variables of temperature and stress and the dependent variable of fatigue life. This model enables precise fatigue life estimation under varying environmental conditions. For instance, the model predicts a 60% reduction in fatigue life when temperature rises from 15 °C to 35 °C at *S* = 0.7, highlighting its utility in material selection for climate-resilient infrastructure, offering a critical tool for optimizing LSAM-50 in pavement design.

Previous research [41] highlights that converting indoor experimental results to real-world pavement applications requires a multiplication factor of 5–700, accounting for parameters including load rest period, crack propagation, lateral load distribution coefficient, and unfavorable seasonal days. Nottingham University studies propose that a correction factor of 77 should be applied at pavement critical state, enabling preliminary estimation of LSAM-50 pavement life via Equation (2):

*N*_P_ = 77 × 10^9.741 − 1.213Lg*ε* − 0.017*T* − 1.579*S*^ (2)

where *N*_P_ denotes LSAM-50 pavement fatigue life (cycle).

Distinct from semi-rigid base asphalt pavements, LSAM-50 flexible base structures exhibit visco-elastoplastic behavior analogous to traditional asphalt mixtures, with pronounced temperature sensitivity [40]. The multi-graded distributions inherent in LSAM-50 pavement necessitate abandonment of the 20 °C standard fatigue temperature used in semi-rigid systems, as this would otherwise result in significant discrepancies between predicted and actual fatigue performance. The fatigue life equations (Equations (1) and (2)) serve dual purposes: optimizing stress-level testing protocols to reduce mixture fatigue testing durations, while also enabling preliminary estimation of LSAM-50 flexible base thickness under diverse climatic conditions during pavement design.

## 4. Conclusions

In this work, the effects of temperature and stress level on the fatigue cracking behavior of LSAM-50 were quantified using DIC. The key findings are as follows:

(1) Laboratory SFT were conducted to investigate the dependency of LSAM-50 fatigue life on temperature and load. Results indicated a progressive reduction in equivalent fatigue life with rising temperature, accompanied by a decelerating rate of decline that closely approximated an exponential decay pattern.

(2) The evolution of horizontal and vertical displacements in LSAM-50 during fatigue cracking was analyzed through a combination of fatigue tests and DIC real-time monitoring. The crack extension process was segmented into three distinct stages: crack initiation, development, and acceleration towards instability, based on the crack extension rate. It was observed that with the increase in temperature (or stress level), the rate of horizontal/vertical displacement development escalates, leading to a decrease in the fatigue life of the material.

(3) Through DIC, the primary crack length, crack density, crack curvature, fractal dimension, and strain, along with the strain cloud, were meticulously measured during the crack propagation phase of LSAM-50 specimens at the range of damage. The findings indicate a direct correlation: as temperature (or stress level) escalates, the primary crack extension rate, crack curvature, and strain in the second stage amplify. Conversely, the crack density and fractal dimension diminish, and the fatigue life is compromised. The stress cloud diagram of LSAM-50 corroborates the onset of stress concentration at the specimen’s extremities, as well as the influence on crack curvature and density.

(4) The correlations between LSAM-50 fatigue life and strain, temperature, and stress level were analyzed. A comprehensive fatigue prediction model was established, accounting for the combined effects of temperature and stress levels, with the equation Lg*N* = 9.741 − 1.213Lg*ε* − 0.017*T* − 1.579*S*, demonstrating an *R*^2^ value of 0.954.

As a visco-elastoplastic material, asphalt mixture is significantly influenced by environmental factors. This investigation focuses solely on the fatigue behavior of LSAM-50 under temperature–load coupling. Laboratory tests predominantly focus on loading frequency to simulate vehicle loads. Notably, future research should explore the variation in crack propagation behavior under diverse loading frequencies and develop corresponding mathematical models. This endeavor will offer robust theoretical underpinnings for material design and optimization.

## Figures and Tables

**Table 1:** Technical properties of asphalt [3].

Test Projects		Measured Values
Penetration (25 °C, 100 g, 5 s) (0.1 mm)		69
Ductility (5 cm/min, 15 °C) (cm)		>100
Ductility (5 cm/min, 10 °C) (cm)		39
Softening point (°C)		47.3
Density of asphalt (g/cm^3^, 15 °C)		1.038
Penetration index (PI)		−0.315
Solubility in trichloroethylene (%)		99.91
Paraffin content (%)		1.6
Dynamic viscosity (Pa·s, 60 °C)		210
Rolling Thin-Film Oven Test (RTFO) (163 °C, 5 h)	Mass loss (%)	−0.1
	Ductility (cm, 10 °C)	7
	Residual penetration ratio (%, 25 °C)	63

**Table 2:** Technical properties of LSAM-50 [3].

Test Projects	Measured Values
Asphalt content (%)	2.9
Density (g/cm^3^)	2.551
Air voids (%)	3.9
Voids in the mineral aggregate (%)	7.8
Voids filled with asphalt (%)	51.3
Compressive strength (MPa, 20 °C)	7.4
Splitting strength (MPa, 20 °C)	1.15
Bending strength (MPa, 20 °C)	3.02

**Table 3:** Main test parameters of SFT [4].

Test Parameter	Test Temperature (°C)	Load Control Mode	Loading Frequency	Stress Level	Waveform
Set value	35, 30, 20, 15, 10, 5, 0, −5, −15	Stress control	10 Hz	0.3, 0.4, 0.5, 0.6, 0.7, 0.8, 0.9	Sinusoidal wave

**Table 4:** Model summary.

Statistical Analysis Parameter	*R*	*R* ^2^	*R*^2^ After Adjustment	Errors in Standard Estimates	Durbin-Watson Value
Calculated value	0.977	0.954	0.951	0.14328	1.310

**Table 5:** Analysis of variance.

Index	Calculated Values of the Following Statistical Analysis Parameters				
	Sum of Squares	Degree of Freedom	Mean Square	*F* Value	Significance
Regression	25.024	3	8.341	406.292	0.000
Residual error	1.211	59	0.021	-	-
total	26.235	62	-	-	-

**Table 6:** Coefficient after statistical analysis.

Argument	Statistical Analysis Coefficients of Different Dependent Variables						
	Unstandardized Coefficient		Standardization Coefficient Beta	*t* Value	Significance	Collinear Statistics	
	*B*	Standard Error				Tolerance	VIF
constant	9.741	1.670	-	5.832	0.000	-	-
Lg*ε*	−1.213	0.480	−0.358	−2.526	0.014	0.039	25.665
*T*	−0.017	0.005	−0.404	−3.341	0.001	0.053	18.713
*S*	−1.579	0.255	−0.489	−6.204	0.000	0.126	7.952

## Data Availability

The original contributions presented in the study are included in the article; further inquiries can be directed to the corresponding author.
